# Forensic Analysis of Human Microbiome in Skin and Body Fluids Based on Geographic Location

**DOI:** 10.3389/fcimb.2021.695191

**Published:** 2021-08-12

**Authors:** Hye-Won Cho, Yong-Bin Eom

**Affiliations:** ^1^Department of Medical Sciences, Graduate School, Soonchunhyang University, Asan, South Korea; ^2^Department of Biomedical Laboratory Science, College of Medical Sciences, Soonchunhyang University, Asan, South Korea

**Keywords:** human microbiome, geography, ethnicity, body fluid, sequencing platform, forensic investigation

## Abstract

High-throughput DNA sequencing technologies have facilitated the *in silico* forensic analysis of human microbiome. Specific microbial species or communities obtained from the crime scene provide evidence of human contacts and their body fluids. The microbial community is influenced by geographic, ethnic, lifestyle, and environmental factors such as urbanization. An understanding of the effects of these external stressors on the human microbiome and determination of stable and changing elements are important in selecting appropriate targets for investigation. In this study, the Forensic Microbiome Database (FMD) (http://www.fmd.jcvi.org) containing the microbiome data of various locations in the human body in 35 countries was used. We focused on skin, saliva, vaginal fluid, and stool and found that the microbiome distribution differed according to the body part as well as the geographic location. In the case of skin samples, *Staphylococcus* species were higher than *Corynebacterium* species among Asians compared with Americans. *Holdemanella* and *Fusobacterium* were specific in the saliva of Koreans and Japanese populations. *Lactobacillus* was found in the vaginal fluids of individuals in all countries, whereas *Serratia* and *Enterobacter* were endemic to Bolivia and Congo, respectively. This study is the first attempt to collate and describe the observed variation in microbiomes from the forensic microbiome database. As additional microbiome databases are reported by studies worldwide, the diversity of the applications may exceed and expand beyond the initial identification of the host.

## Introduction

Microorganisms are ubiquitous on all terrestrial and aquatic environments. Microbes have played an important role in medicine, fermentation, and food industry for millennia but have not generally been exploited in forensic medicine (Alan and Sarah, 2012). However, molecular biology tools such as DNA fingerprinting, whole genome sequencing, and microarray analysis have significantly advanced the field of microbial forensics over the last two decades (Cummings and Relman, 2002; Li et al., 2012; Kuiper, 2016; Schmedes and Budowle, 2019). Massively parallel sequencing (MPS) was applied to the routine analysis of microbial forensic evidence (Turnbaugh et al., 2007; Human Microbiome Project Consortium, 2012). MPS, which is also known as next-generation sequencing (NGS), enables the detection of low levels of microorganisms and unknown pathogens even in mixed samples (Schmedes et al., 2016; Singh and Kaur, 2020). With the reduction in sequencing costs and the continued development of bioinformatics analysis *via* increased throughput, MPS has been used to characterize the microbial community for forensic applications (Hachem et al., 2020).

Forensic analysis using microbiome data obtained from suspects, victims, and the environment has the potential to support crime scene investigations (Clarke et al., 2017). The microbiome data can be used as potential evidence for human identification (Woerner et al., 2019), detection of body fluids (Dobay et al., 2019), and geographic location of individuals (Grantham et al., 2019). The human microbiome can be collected from locations such as human skin (Schmedes et al., 2018) and body fluids (Dobay et al., 2019), houses (Song et al., 2013), dorm rooms (Luongo et al., 2017), classrooms (Kembel et al., 2014), offices (Chase et al., 2016), bathrooms, (Flores et al., 2011), and personal belongings like shoes (Lax et al., 2015), keyboards (Lee et al., 2015), fabrics (Lee et al., 2016), and cellphones (Lax et al., 2015). Body fluids and skin swabs collected from individual populations exhibit specific characteristics. The present review shows descriptions of forensic indicator sites, including skin, vaginal fluid, stool, and saliva from populations in diverse geographical locations. Sequencing of the microbiome obtained from diverse locations demonstrated that bacterial DNA can be used to indicate individual lifestyle and behavioral patterns (Clarke et al., 2017). Thus, microbiomes provide geographic, ethnic, or lifestyle information that can be used by forensic scientists to identify the origin of unknown samples during criminal investigation. Also, evidence suggests that the combination of microbiology, molecular biology, and forensic investigations may enable the identification of the suspect and the victim based on human microbiome samples found at a crime scene, especially when traditional sources of human DNA are insufficient (Richardson et al., 2019; Phan et al., 2020).

The differences between individuals can be determined based on single sample studies; however, the forensic examination is limited by the sample size and scope, such as the availability of only one metadata variable or location (Singh et al., 2021). In this study, the Forensic Microbiome Database (FMD) (http://www.fmd.jcvi.org) was used to obtain the comprehensive microbiome data of various body sites. FMD represents a database containing the microbiomes from 35 countries (139 cities) and links publicly available 16S rRNA gene-derived taxa with their geographic origins, often at the level of the city (Clarke et al., 2017). Since an individual’s microbiome is in part influenced by geography, diet, ethnicity, and degree of urbanization, the knowledge of specific microbiome composition in a population and/or environment can facilitate the utilization of such data in determining the host’s geographical location. In addition, since crime can be transnational, the geographical location determined using the microbiome data can facilitate forensic investigations. Our review represents a timely contribution to state-of-the-art forensic investigatory tools used to analyze crime scene investigations.

## Forensic Applications of Microbiome

Microbiome analysis has been utilized in human health and disease as well as forensic science aided by advanced computational tools in bioinformatics analysis, using refined methodology, sampling, and library preparation (Robinson et al., 2021). The use of microbiome analysis from different sets of body sites and diverse environments, specifically in the forensic field, can be used to establish the geographic location of criminal events and determine the cause of death and the identity of individuals (Clarke et al., 2017; García et al., 2020). For instance, skin microbiomes are unique to individuals, and thus used to determine personal identity. Also, the distinct microbiome of individual body sites provides valuable evidence in criminal investigations, such as sexual assaults (Schmedes et al., 2017). Meanwhile, specific microorganisms such as *Helicobacter pylori* can be linked to a host or environment since they are functionally unique with a distinct composition based on geographic location (Kersulyte et al., 2010; Nagasawa et al., 2013). Thus, the diverse location across different climate zones can also be accurately explained by the microbiome and can be used to predict the suspect’s identity (Chase et al., 2016). A recent study even identified the urban microbiomes and antimicrobial resistance genes, which reflect the significant features of cities, and highlighted their forensic applications (Danko et al., 2021).

The field application of forensic microbiome analysis requires the evaluation of its effectiveness and validation of its stability and accuracy. In addition, many basic studies are needed to demonstrate the potential role of microbial community profiles in the forensic application. Therefore, forensic microbiome analysis is indispensable as microevidence although additional studies and evaluation of techniques used in forensic microbiome analysis are required, under circumstances where criminals leave no fingerprints, blood, saliva, semen, or other bodily fluids that can be analyzed using the extracted DNA samples (Eom, 2018).

Accordingly, the stability of the microbial community over time or with translocation is a distinct advantage that cannot be overlooked in microbial forensics. Indeed, the forensic application of microbiome is strongly linked to the stability of the microbiome (Tozzo et al., 2020). The microbiome collected by swab remains stable until 2 weeks under typical indoor conditions (Fierer et al., 2010). Individuals can be distinguished based on their microbial profiles, especially gut microbiome even up to a year (Franzosa et al., 2015). Analysis of bacterial translocation has been mostly used to define uncertain causes of death. The swift translocation of intestinal bacteria is triggered immediately after death and can be used to distinguish contaminants from established microbiota. Heimesaat et al. suggested that the kinetics of *Enterobacteriaceae* and *Enterococcus* can serve as an indicator to interpret the time of death (Heimesaat et al., 2012). Also, the translocation of *Clostridium* in the internal organs of humans postmortem was identified (Tuomisto et al., 2013; Javan et al., 2016; García et al., 2020). The critical role of forensic microbiology in the prevention of unresolved issues and analysis of evidence beyond a reasonable doubt will increase. In this review, we will discuss the five most researched themes: skin, saliva, vaginal fluid, stool, and sequencing platforms.

## The Forensic Microbiome Database

This study was accomplished using the FMD described at http://www.fmd.jcvi.org and reported in a recent study (Singh et al., 2021). In brief, the FMD contains publicly available 16S rRNA gene sequence data obtained from multiple body sites (Clarke et al., 2017). The FMD sequence data are derived from samples obtained from healthy adults (≥18 years) across multiple body sites based on 95 projects with 79 PubMed references. Also, the FMD project additionally incorporated oral and stool samples derived from a total of 161 women from Barbados, Santiago, Pretoria, and Bangkok. Sequence data were also downloaded from public websites including NCBI SRA (https://www.ncbi.nlm.nih.gov/sra), EBI (https://www.ebi.ac.uk/), and MG-RAST (https://www.mg-rast.org/), which were all accessed on July 13, 2021 and then analyzed (Singh et al., 2021). The collected data were analyzed in the following order. Using the UPARSE pipeline, the taxonomic population distribution for each sequence from the public dataset was analyzed with the FMD. Using machine learning techniques, the bacterial taxa, which can be best distinguished by geographical location, were identified. In summary, the FMD data include body site location, microbial count, and geographic information (country, subdivision, i.e., the state or department or province, and city). A complete list of available data, including studies, populations, and counts, is found online (http://www.fmd.jcvi.org). The publicly available FMD will attract additional data. The forensic microbiome analyses will be strengthened by the inclusion of further microbiome data from various studies worldwide. A review of FMD based on other studies included in this review may motivate other investigators interested in using the FMD data and expanding the database.

## Skin

The healthy skin microbiome exhibits substantial taxonomic diversity, which is dependent on both geography and individuality (Oh et al., 2014). Skin bacteria are affected by age, gender, and external environmental factors, including moisture, temperature, and geographical location. Skin microbial signatures may remain for protracted periods on surfaces or objects in contact with humans (Gupta et al., 2017). Thus, the skin, which is the largest organ of the human body, contains significantly higher bacterial, viral, and fungal diversity than in other locations such as the gut, blood, or saliva (Oh et al., 2014). The diversity of skin microbiome depends on the taxonomic depth of the bioinformatics analysis and the discriminating power. Thus, while it is possible to define the various populations based only on the bacterial phylum, the analysis of microbial community is comparable with DNA-based analysis if it is performed at the level of genus and species (Tozzo et al., 2020). Accordingly, the structure of the human skin microbiome is unique to the individual (Flores et al., 2014). Furthermore, it was suggested that the skin microbiome can be obtained readily from the crime scene providing an alternative line of investigation (Neckovic et al., 2020).

**Figure 1:**
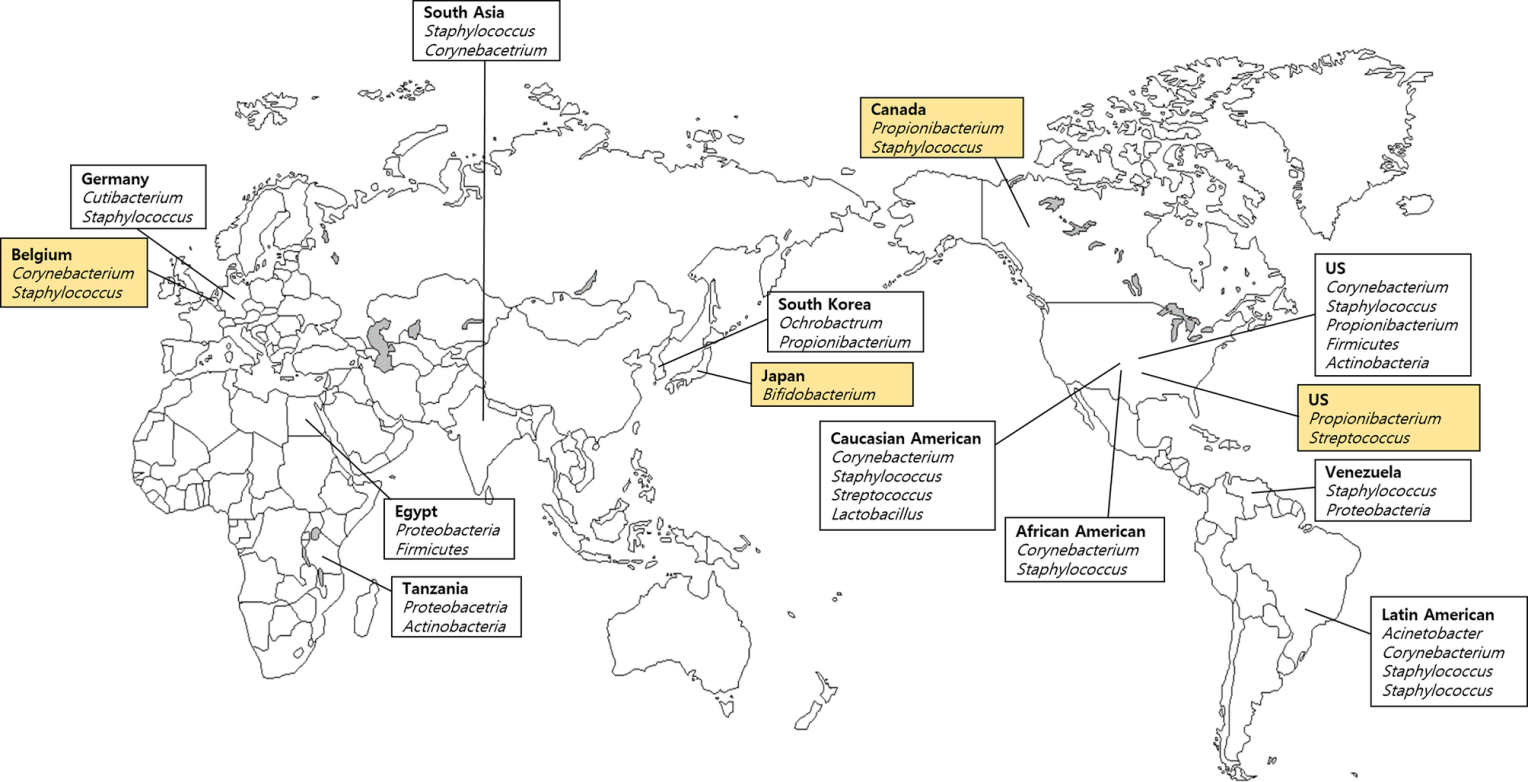
Geographical variation of skin bacteria based on the Forensic Microbiome Database (FMD) (www.fmd.jcvi.org) data and non-FMD. The yellow-colored box is from the FMD data, and only bacteria which account for over 10% are shown. Four countries from the FMD and 10 results from previous studies were included, and the microbiome profiling was generated using 16S rRNA gene sequencing.

We analyzed four skin samples from four different countries listed in the FMD (**Table 1**), and 16 samples from 10 countries, which were not included in the FMD (**Table 2**). A previous study reported the relative abundance of taxa on the hand microbiome and identified four main phyla (Firmicutes, Actinobacteria, Proteobacteria, and Bacteroidetes) (Edmonds-Wilson et al., 2015). They also reported that most individuals carried between eight and 24 families of bacteria dwelling on their hands, including the major taxa (*Staphylococcaceae*, *Corynebacteriaceae*, *Propionibacteriaceae*, and *Streptococcaceae*) (Edmonds-Wilson et al., 2015). Above all, *Propionibacteriaceae*, which is relatively higher in abundance, and *Cutibacterium acnes* (formerly *Propionibacterium acnes*), are both established commensal bacteria found in the skin, similar to *Staphylococcus*. Also, *C. acnes* resides at all body sites, with relative abundances ranging from 35% to 89% (Schmedes et al., 2017). These data suggest that *C. acnes* may play an informative role in forensic investigation using skin microbiomes. Even though a core microbiome was observed on the hand, pronounced intra- and interindividual differences in bacterial community composition were observed (Siqueira et al., 2012). Based on FMD data, *Propionibacterium* and *Streptococcus* dominate the microbial community in the United States, representing typical hand microbial clusters (**Figure 1**). However, although expected to be ubiquitous among humans, *Propionibacterium* was not detected in most hand microbial samples in South Korea and Yokohama (Japan) (**Table 1**).

**Table 1:** Distribution of dominant bacteria (over 10%) by countries of four body areas including skin, saliva, vagina, and stool from the Forensic Microbiome Database (FMD).

Sample	Country	Dominant bacteria	Note	Sample size (*N*)
Skin	Belgium	*Corynebacterium*, *Staphylococcu*, *Moraxella*	Ghent	9
	Japan	*Bifidobacterium*	Yokohama	29
	USA	*Streptococcus*, *Propionibacterium*	Boulder	76
	Canada	*Bacillus*, *Streptococcus*, *Propionibacterium*	Waterloo	1
Saliva	India	*Streptococcus*	Paderu	9
		*Streptococcus*	Guwahati	10
		*Streptococcus*, *Veillonella*, *Prevotella*	Jammu	12
		*Streptococcus*, *Porphyromonas*	Dhanbad	9
		*Streptococcus*, *Veillonella*, *Prevotella*	Tamil Nadu	10
		*Streptococcus*, *Porphyromonas*	Telangana	12
		*Streptococcus*, *Prevotella*	Uttarakhand	11
		*Streptococcus*	West Bengal	14
	Italy	*Streptococcus*, *Prevotella*, *Neisseria*	Trento	13
	Japan	*Fusobacterium*, *Collinsella*	Yokohama	23
		*Collinsella*, *Granulicatella*	Fukuoka	177
		*Bifidobacterium*, *Blautia*	Hisayama	2,370
	South Korea	*Holdemanella*, *Streptococcus*, *Neisseria*, *Bifidobacterium*	Sudogwon	103
	USA	*Haemophilus*, *Streptococcus*, *Veillonella*, *Campylobacter*	Minnesota_Mankato	3
		*Streptococcus*, *Veillonella*	Missouri	116
		*Streptococcus*	Ohio_Columbus	25
		*Streptococcus*, *Veillonella*, *Prevotella 7*	Oklahoma_Tulsa	1
		*Haemophilus*, *Streptococcus*, *Neisseria*	Texas_Austin	5
		*Haemophilus*, *Streptococcus*, *Veillonella*	Texas_Houston	240
Vagina	Australia	*Bifidobacterium*		67
	Italy	*Bifidobacterium*		16
	South Africa	*Bifidobacterium*		131
Vaginal introitus-mid Vagina	USA	*Lactobacillus*	Minnesota	2
		*Lactobacillus*	Missouri	88
		*Lactobacillus*	Oklahoma	2
		*Lactobacillus*	Texas	105
Stool	Argentina	*Bacteroides*	Rosario	39
	Australia	*Bacteroides*	Brisbane	39
	Belgium	*Bacteroides*	Ghent	45
	Burkina Faso	*Streptococcus*	Boulpon	2
	Canada	*Bacteroides*	Montreal	35
		*Bacteroides*	Nunavut	49
	Chile	*Bacteroides*, *Prevotella*	Santiago	109
	China	*Bacteroides*, *Bifidobacterium*, *Faecalibacterium*	Beijing	27
		*Bacteroides*, *Bifidobacterium*	Lanzhou	23
		*Prevotella*, *Phascolarctobacterium*	Baise	15
		*Phascolarctobacterium*, *Blautia*	Nanning	16
		*Phascolarctobacterium*, *Blautia*	Harbin	21
		*Phascolarctobacterium*	Zhengzhou	18
		*Bacteroides*, *Phascolarctobacterium*	Hohhot	19
		*Bacteroides*, *Phascolarctobacterium*	Xilinguole	21
		*Phascolarctobacterium*	Wuxi	18
		*Phascolarctobacterium*, *Blautia*, *Megamonas*	Chengdu	11
		*Phascolarctobacterium*, *Prevotella*	Lhasa	10
		*Phascolarctobacterium*, *Prevotella*	Nagqu	26
		*Phascolarctobacterium*	Altay	3
		*Phascolarctobacterium*	Urumqi	13
		*Phascolarctobacterium*, *Megamonas*	Yili Prefecture	6
		*Phascolarctobacterium*, *Megamonas*	Dali Bai Autonomous Prefecture	250
		*Megamonas*, *Prevotella*	Kunming	12
	Gambia	*Prevotella*, *Subdoligranulum*	Banjul	125
	Germany	*Akkermansia*, *Bacteroides*	Goettingen	15
	Ghana	*Akkermansia*, *Bacteroides*, *Subdoligranulum*, *Ruminococcus*	Eikwe	5
	India	*Prevotella*	Baksa	5
		*Prevotella*	Golaghat	5
		*Prevotella*	Karbi anglong	5
		*Prevotella*	Tinsukia	10
		*Prevotella*	Imphal East	1
		*Prevotella*	Imphal West	3
		*Prevotella*	Senapati	5
		*Prevotella*	Ukhrul	5
		*Prevotella*	East Sikkim	8
		*Prevotella*	North Sikkim	1
		*Prevotella*	South Sikkim	4
		*Faecalibacterium*	West Sikkim	1
		*Prevotella*	Vellore	35
		*Prevotella*	Adilabad	9
		*Prevotella*, *Succinivibrio*	Khammam	7
	Indonesia	*Prevotella*	Bali	19
		*Prevotella*	Yogjakarta	28
		*Clostridium sensu stricto*	Medan	6
	Ireland	*Bacteroides*, *Bifidobacterium*	Cork	981
	Italy	*Bifidobacterium*, *Faecalibacterium*, *Subdoligranulum*	Bologna	16
		*Bacteroides*	Rome	39
		*Bifidobacterium*, *Faecalibacterium*	Milan	54
		*Pseudobutyrivibrio*	Florence	1
	Japan	*Bifidobacterium*	Aichi	19
		*Bifidobacterium*	Mie	11
		*Bifidobacterium*	Shizuoka	17
		*Bifidobacterium*	Ehime	34
		*Bifidobacterium*	Okayama	14
		*Bifidobacterium*	Hokkaido	38
		*Bifidobacterium*	Kanakawa	45
		*Bifidobacterium*, *Porphyromonas*, *Bacteroides*	Yokohama	11
		*Bifidobacterium*	Hyogo	10
		*Bifidobacterium*	Kyoto	13
		*Bifidobacterium*	Osaka	25
		*Bifidobacterium*, *Succinivibrio*	Chiba	33
		*Bifidobacterium*, *Fusicatenibacter*	Gunma	13
		*Bifidobacterium*	Ibaraki	21
		*Bifidobacterium*	Kanagawa	34
		*Bifidobacterium*	Saitama	35
		*Bifidobacterium*, *Fusicatenibacter*	Tochigi	19
		*Bifidobacterium*	Tokyo	362
		*Bifidobacterium*	Fukuoka	59
		*Bifidobacterium*	Kagoshima	20
		*Bifidobacterium*, *Fusicatenibacter*	Nagasaki	13
		*Bifidobacterium*	Saga	51
		*Bifidobacterium*	Miyagi	40
	Malawi	*Bacteroides*	Mbiza	1,038
	Philippines	*Bifidobacterium*, *Faecalibacterium*	Baybay	11
		*Bifidobacterium*, *Faecalibacterium*	Ormoc	14
	Spain	*Bacteroides*, *Prevotella*	Barcelona	123
	Sweden	*Prevotella*, *Blautia*, *Faecalibacterium*	Stockholm	2
		*Faecalibacterium*, *Bifidobacterium*	Uppsala	2
	Taiwan	*Bacteroides*, *Bifidobacterium*	Taipei	46
	Tanzania	*Faecalibacterium*	Dedauko	15
		*Faecalibacterium*	Sengele	5
	Thailand	*Bacteroides*, *Prevotella*	Bangkok	93
		*Prevotella*, *Faecalibacterium*	Khon Kaen	25
	Uganda	*Bifidobacterium*, *Prevotella*, *Bacteroides*	Kampala	50
	UK	*Bacteroides*	London	2,644
		*Bacteroides*, *Faecalibacterium*	Newcastle	20
	USA	*Bacteroides*	Los Angeles	9
		*Bacteroides*	Pal Alto	21
		*Blautia*, *Ruminococcus*	Boulder	1
		*Bacteroides*, *Faecalibacterium*, *Prevotella*	Boston	15
		*Bacteroides*	Canbridge	169
		*Bacteroides*	Mankato	4
		*Bacteroides*	Columbia	2
		*Bifidobacterium*, *Ruminococcus*	St. Louis	1,674
		*Bifidobacterium*	New York	32
		*Bacteroides*, *Prevotella*	Lancaster	606
		*Bacteroides*, *Parabacteroides*	Philadelphia	1
		*Bacteroides*	Austin	5

**Table 2:** Summary of country in non-FMD studies assessing microbial composition of the skin, saliva, vaginal fluid, and stool.

Sample	Country	Dominant bacteria	Sample size	Demographics (race, gender, age)	Sampling collection	Medical history	Statistical analysis	Note	Reference
**Skin**	South Korea	*Ochrobactrum*, *Propionibacterium*	3	Over 20	Fabric	Healthy	Chao 1, Shannon diversity index, XOR analysis	Hand	Lee et al. (2016)
	South Asia	*Staphylococcus*, *Acinetobacter*, *Corynebacetrium*	20	India, mean of age = 37.9	Swab	Healthy	Linear discriminant analysis effect size (LEfSe), UCLUST	Arm	Perez et al. (2016)
		*Staphylococcus, Corynebacterium, Anaerococcus*	20	India, mean of age = 37.9	Swab	Healthy	LEfSe, UCLUST	Axilla	Perez et al. (2016)
	Germany	*Cutibacterium*, *Staphylococcus*	7	Males, between 18 and 60 years	Swab	Healthy	Bioconductor workflow	Antecubital fossa	Francuzik et al. (2018)
	**USA**	*Corynebacteria*, *Staphylococcus*	10	Males and females, 20–41 years	Swab	Healthy	Shannon diversity index, distance-based OTU and richness (DOTUR)	Antecubital fossa	Grice et al. (2009)
	USA	*Propionibacterium*	4	28–55 years	Swab	Healthy	Chao 1, UCLUST, UniFrac	Forearm	Blaser et al. (2013)
	USA	Firmicutes, Actinobacteria	15 (13 were white and of European ancestry, and 2 were Chinese-American)	Females, graduate student	Glove juice method	Healthy	UniFrac (principal coordinate analyses (PCoA))	Hand	Hospodsky et al. (2014)
	Caucasian America (US borne, of European ancestry)	*Corynebacterium*, *Staphylococcus*, *Streptococcus*, *Lactobacillus*	16	Mean of age = 27.6	Swab	Healthy	LEfSe, UCLUST	Arm	Perez et al. (2016)
		*Corynebacterium*, *Staphylococcus*	16	Mean of age = 27.6	Swab	Healthy	LEfSe, UCLUST	Axilla	Perez et al. (2016)
	African America	*Corynebacterium*, *Staphylococcus*	18	Mean of age = 25.9	Swab	Healthy	Linear discriminant analysis effect size (LEfSe), UCLUST	Arm	Perez et al. (2016)
		*Corynebacterium*, *Staphylococcus*	18	Mean of age = 25.9	Swab	Healthy	LEfSe, UCLUST	Axilla	Perez et al. (2016)
	Latin America	*Acinetobacter*, *Corynebacterium*, *Staphylococcus*	20 (Ecuador = 15 and Mexico = 5)	Ecuador and Mexico	Swab	Healthy	LEfSe, UCLUST	Arm	Perez et al. (2016)
		*Staphylococcus*	20 (Ecuador = 15 and Mexico = 5)	Ecuador and Mexico	Swab	Healthy	LEfSe, UCLUST	Axilla	Perez et al. (2016)
	Venezuela (Amerindian)	*Staphylococcus*, Proteobacteria	72	2 months–80 years	Swab	Healthy	Chao 1, UCLUST, UniFrac	Forearm	Blaser et al. (2013)
	Tanzania	Proteobacteria, Actinobacteria	29	Females, graduate student	Glove juice method	Healthy	UniFrac	Hand	Hospodsky et al. (2014)
	Egypt	Proteobacteria, Firmicutes	5		Swab	Healthy		Antecubital fossa	Ramadan et al. (2016)
**Saliva**	South Korea	*Neisseria*	543 (198 males, 345 females)	Males and females, 40–79 years	Chew gum and stimulated saliva samples	Orally healthy	UCLUST, UniFrac, Shannon diversity index		Takeshita et al. (2014)
	Japan	*Prevotella*, *Veillonella*	2,272 (1,011 males, 1,261 females)	Males and females, 40–79 years	Chew gum and stimulated saliva samples	Orally healthy	UCLUST, UniFrac, Shannon diversity index		Takeshita et al. (2014)
	China	*Haemophilus, Prevotella*	29	Males and females, 20–40 years	5 ml of spontaneous, whole, unstimulated saliva	Orally healthy, Normal weight group (BMI = 18.5–20)	RDP classifier, Chao 1, Shannon diversity index, UniFrac, Kurskal–Wallis test		Wu et al. (2018)
	Qatari	*Prevotella*	997 (442 males and 555 females)	Males and females, 18≤ years	5 ml of spontaneous, whole, unstimulated saliva	Healthy and unhealthy	QIIME, LEfSe		Murugesan et al. (2020)
	Thailand	*Streptococcus*, *Veillonella*	50 (27 males and 23 females)	Males and females, 7–15 years	Chew gum and stimulated saliva samples	Orally healthy	Shannon diversity index, UCLUST, UniFrac		Mashima et al. (2019)
	Swiss	*Streptococcus*, *Neisseria*, *Prevotella*	2	Over 20	5 ml of spontaneous, whole, unstimulated saliva	Orally healthy	CD-HIT-EST, DESeq		Leake et al. (2016)
	Germany	Firmicutes, Proteobacteria, Bacteroidetes	10	20–40 years	Volunteer spit up to 2 ml of saliva into tubes containing 2 ml lysis buffer	Orally healthy	Shannon diversity index, Sørensen index, UniFrac		Li et al. (2014)
	**Bolivia**	*Serratia*	10	Males and females	Volunteer spit up to 2 ml of saliva into tubes containing 2 ml lysis buffer	Orally healthy	DISTLM, UniFrac, SeqMatch		Nasidze et al. (2009a)
	**Congo**	*Enterobacter*	10	Males and females	Volunteer spit up to 2 ml of saliva into tubes containing 2 ml lysis buffer	Orally healthy	DISTLM, UniFrac, SeqMatch		Nasidze et al. (2009a)
	Africa	Firmicutes, Proteobacteria	66 (Democratic Republic of Congo = 15, Sierra Leone = 13, Uganda = 38)	Democratic Republic of Congo, Sierra Leone, Uganda, males and females, 20–40 years	Volunteer spit up to 2 ml of saliva into tubes containing 2 ml lysis buffer	Orally healthy	UniFrac, Shannon diversity index, Sørensen index		Li et al. (2014)
	Alaska	Firmicutes, Proteobacteria, Bacteroidetes, Actinobacteria	76	Four native Alaskan communities, males and females, 20–40 years	Volunteer spit up to 2 ml of saliva into tubes containing 2 ml lysis buffer	Orally healthy	UniFrac, Shannon diversity index, Sørensen index		Li et al. (2014)
**Vaginal fluid**	**South Korea**	*L. crispatus*, *L. fermentum*, *L. jensenii*, *L. salivariusm*	110		Vaginal swab	Healthy	Clustal X, TreeExplorer		Jin et al. (2007)
	Japan	*L. crispatus*, *L. iners*	73	3 age groups (18–25, 26–34, and 35–45 years)	Vaginal swab				Zhou et al. (2010)
	**India**	*L. jensenii*	199		Vaginal swab	Healthy		Southwest	Pramanick et al. (2017)
		*L. reuteri*, *L. fermentum*, *L. salivarius*	80	18–45 years	Vaginal swab	Healthy	RAPD analysis	Central	Garg et al. (2009)
		*L. crispatus*, *L. gasseri*, *L. jensenii*	132	18–45 years	Vaginal swab	Healthy	SAS Institute	South	Madhivanan et al. (2015)
		*L. mucosae*, *E. faecalis*	69	18–35 years	Vaginal swab	Healthy pregnant and nonpregnant	ClustalX, BLASTn, EZ-Taxon	Northeast	Das Purkayastha et al. (2019)
	Belgium	*L. crispatus*, *L. iners*, *L. jensenii*, *B. xylanisolvens*, *B. thetaiotaomicron*, *B. fragilis*	19	25–39 years	Brush™ IUMC Endometrial Sampler	Healthy premenopausal	PRIMER		Verstraelen et al. (2016)
	Swiss	*L. crispatus*, *L. iners*, *L. gasseri*, *L. jensenii*	23		Vaginal swab	Healthy	RAPD analysis		Vasquez et al. (2002)
	**Europe**	*L. crispatus*, *L. iners*	416	18–44 years	Vaginal swab	Healthy	Inverse Simpson’s index, Bray–Curtis method, LEfSe		Fettweis et al. (2014)
	North America	*L. iners*	396	12–45 years	Vaginal swab	Healthy and not pregnant	RDP Naïve Bayesian Classifier		Ravel et al. (2011)
	**African America**	*L. iners*, *G. vaginalis*	1,268	18–44 years	Vaginal swab	Healthy	Inverse Simpson’s index, Bray–Curtis method, LEfSe		Fettweis et al. (2014)
	Uganda	*L. reuteri*, *L. crispatus*, *L. vaginalis*, *L. gasseri*	250		Vaginal swab	Healthy	Clustal X, TreeExplorer		Jin et al. (2007)
	Republic of South Africa	*L. crispatus*	40	18–44 years	Vaginal swab	Healthy (premenopausal and HIV uninfected)	GraphPad Prism 4 software		Pendharkar et al. (2013)
**Stool**	**South Korea**	Bacteroidetes, Firmicutes, Proteobacteria	8	Females, over 65		Healthy	Ion Reporter™		Shin et al. (2016)
	Japan	*Actinobacteria*, *Bifidobacterium*, *Clostridium*	13			Healthy	TaxCollector, UPGMA, UniFrac		Nam et al. (2011)
	China	*Bacteroides*	1			Healthy	TaxCollector, UPGMA, UniFrac		Nam et al. (2011)
	India	*Prevotella*, *Faecalibacterium*	80	Males and females, 18–55 years		Healthy and whose body mass index (18.50 to 30.00 kg/m^2^) with minimum of 45 kg weight		West	Tandon et al. (2018)
	**Turkey**	*Prevotella*	436 (212 males and 224 females)	Males and females, 18–70 years		Healthy	Shannon diversity index, Simpson, Chao		Deschasaux et al. (2018)
	Himalaya	*Treponema*, *Prevotella*	56	Males and females, 18≤ years		Healthy and unhealthy	Shannon diversity index, Simpson, phyloseq, SparCC		Jha et al. (2018)
	Netherlands	*Clostridiales*	1,328 (695 males and 633 females)	Males and females, 18–70 years		Healthy	Shannon diversity index, Simpson, Chao		Deschasaux et al. (2018)
	USA	Firmicutes	41			Healthy	TaxCollector, UPGMA, UniFrac		Nam et al. (2011)
	Morocco	*Prevotella*	605 (324 males and 281 females)	Males and females, 18–70 years		Healthy	Shannon diversity index, Simpson, Chao		Deschasaux et al. (2018)
	African Surinamese, South Asian Surinamese	*Bacteroides*	1,703 (731 males and 972 females)	Males and females, 18–70 years		Healthy	Shannon diversity index, Simpson, Chao		Deschasaux et al. (2018)

The vaginal samples were obtained from women only, and stool samples were collected with stool collection tubes of participants, respectively.

Additionally, in a comparative analysis of taxa associated with hand microbiomes in Americans and South Koreans, the American sample showed the highest proportion of *Propionibacterium* (31.8%) and *Streptococcus* (18.3%) (**Table 1**). In contrast, in South Korea, Lee et al. (2016) reported a preponderance of *Ochrobactrum* (40.0%) and *Propionibacterium* (30.4%) (**Table 2**). The samples also revealed *Staphylococcus* (7.1%). The palm of hand microbiome in Canada was predominantly composed of *Bacillus* (27.7%), *Streptococcus* (22.5%), and *Propionibacterium* (12.7%) (**Table 1**). Apparent differences existed between North American and Asian populations. *Ochrobactrum* was not detected in American and Canadian populations, whereas only a few species of *Streptococcus* were found in South Korean samples, which were even higher in American and Canadian samples. Hospodsky et al. (2014) analyzed American and Tanzanian hand microbiomes and reported differences compared with the US hand microbiome data (FMD). *Propionibacteriaceae*, *Staphylococcaceae*, and *Streptococceaceae* families were predominantly detected in American hand samples, and soil-associated *Rhodobacteraceae* and *Nocardiodaceae* were the most dominant on Tanzanian hands. At the phylum level, Firmicutes and Proteobacteria were the most abundant in American and Tanzanian hand microbiomes, respectively (Hospodsky et al., 2014).

Byrd et al. (2018) described skin microbial communities at four body sites, including forehead, antecubital fossa, forearm, and foot in the American samples. They reported that forearms exhibited an abundance of *Propionibacterium* and *Staphylococcus*. However, *Propionibacterium* and *Staphylococcus* were not the dominant genera on the Japanese forearms. According to the FMD, Japan (Yokohama) showed *Bifidobacterium* (27.7%) predominantly in the forearm microbiome. Two cities (Yokohama and Tokyo) with forearm microbiome data were mentioned in the FMD, and fewer proportions of bacteria were detected in Tokyo (*Streptococcus* 6.52% and *Moraxella* 3.49%). Also, the two cities did not share bacteria. Blaser et al. (2013) reported differences in the forearm microbiota between the American and the two groups of Amerindians in Venezuela (Blaser et al., 2013). *Staphylococcus* was the dominant genus, followed by *Propionibacterium*, *Streptococcus*, *Pseudomonas*, and *Corynebacterium.* Accordingly, they proposed a profound association between microbiota and modernization, ancestry, and biogeography (Blaser et al., 2013).

Furthermore, Perez et al. (2016) analyzed the forearm microbiome of six ethnic groups (Caucasian-American, African-American, African, Latin American, East Asian, and South Asian) living in the USA. In that study, most groups comprised Actinobacteria and Firmicutes at the phylum level and *Staphylococcus* and *Corynebacterium* at the genus level. Among the six groups, Latin Americans, in particular, showed a relative abundance of Proteobacteria and *Acinetobacter* (Perez et al., 2016). Notable differences in microbiome were found between ethnicities in line with Blaser et al. (2013).

In the case of the antecubital fossa, no apparent differences were found between left and right antecubital fossa in the American samples (**Table 1**). *Propionibacterium* dominated both left and right antecubital fossae (41.1% and 44.1%, respectively), followed by *Staphylococcus* (12.9% and 11.8%, respectively). Other studies reported that *Corynebacteria* species dominated the antecubital fossa, as well as moist sites such as nares (inside the nostril) and axillary vault, although *Staphylococci* species were sparsely detected in the American population (Grice et al., 2009; Byrd et al., 2018). However, Egyptians differed slightly from the Americans in that Proteobacteria were the predominant phyla in all Egyptian samples of the antecubital fossa (57%), followed by Firmicutes (36%) and Actinobacteria (4%) (Ramadan et al., 2016). In addition, the antecubital fossa microbiome in healthy Germans contained *C. acnes* and *Staphylococcus epidermidis* as the dominant bacteria (46.9% and 10.5%, respectively) (Francuzik et al., 2018). Each country exhibited characteristic microorganisms in the antecubital fossa compared with other body sites.

Based on these studies, it is conceivable that skin microbial communities vary even within the same country, based on topography and lifestyle. However, the diversity may be attributed to analytical differences or a combination of analytical differences and environmental factors. Nonetheless, the data are promising and suggest that microbiomes can be distinguished based on geographical location, ethnicity (perhaps largely affected by geography), and lifestyle factors. A more standardized protocol is required to characterize the skin microbiomes from diverse population samples to determine the factors contributing to the observed variation and the occasional differences within the population.

## Saliva

Saliva is another body fluid found at crime scenes (Virkler and Lednev, 2009). Saliva can be found on a variety of specimens left behind at the crime scene, including cigarettes, vaping tools, bottles, cups, victim’s skin, bite marks, lip prints, and drug paraphernalia (Kapoor and Chowdhry, 2018). Accepted, presumptive, and confirmatory tests of forensic samples based on saliva are available (Old et al., 2009; Virkler and Lednev, 2009). However, some of these tests, particularly the presumptive tests, are often not specific to saliva. Other tests cannot differentiate vaginal fluid from saliva which may serve as a significant piece of evidence in reconstructing cases related to sexual assault. In addition, saliva may be detected in trace levels and the protein of interest for these tests may not be adequate enough. However, saliva contains microorganisms, which facilitates forensic investigations when combined with longstanding salivary biomarkers (Leake et al., 2016).

**Figure 2:**
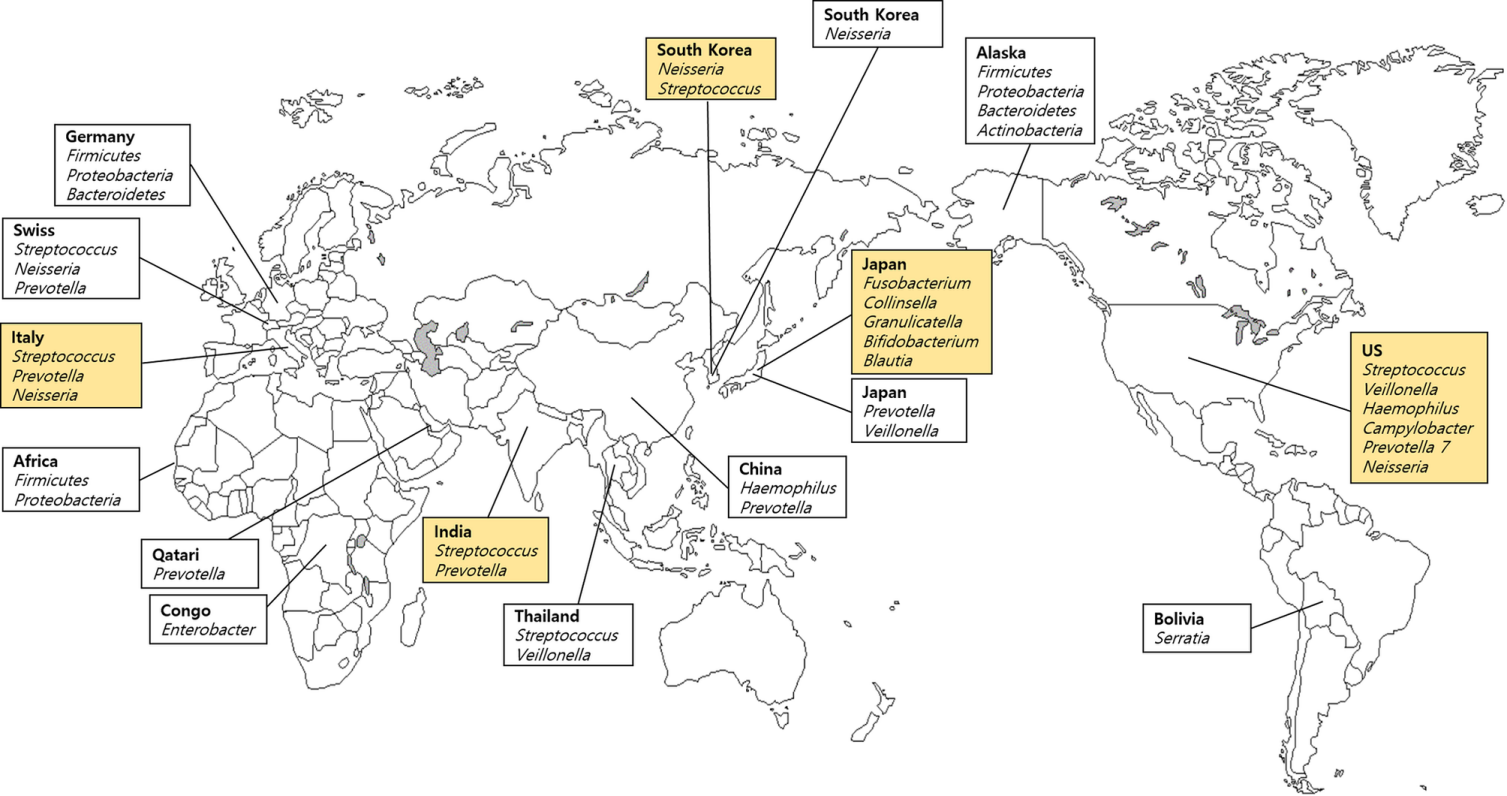
Dominant bacteria in saliva samples based on the Forensic Microbiome Database (www.fmd.jcvi.org) data and non-FMD studies. The yellow-colored box is from the FMD data, and only bacteria which account for over 10% are shown. Five countries from the FMD and 11 countries from previous studies were included, and the microbiome profiling was generated using 16S rRNA gene sequencing.

We included 19 samples of saliva obtained from five different countries listed in the FMD (**Table 1**), and 11 samples from each of the 11 countries, which are not included in the FMD (**Table 2**). Different geographical and environmental factors, such as diet, elements of hygiene, humidity, climate, temperature and oral disease, can affect the composition of microbial communities (Li et al., 2014). Several studies of saliva have demonstrated a potential geographic signature involving the oral microbiome (Nasidze et al., 2009a; Clarke et al., 2017). Saliva samples obtained from India, Italy, Japan, South Korea, and the USA were used in the FMD data. *Streptococcus* were the main bacteria in samples derived from most countries (**Figure 2**). *Rothia* was the highest in prevalence among Japanese; *Prevotella* was the highest in India and Italy; and *Neisseria* was the predominant salivary microbe among Italians and South Koreans. Takeshita et al. (2014) analyzed healthy South Koreans and Japanese and found that the salivary microbiome of Koreans harbored higher proportions of *Neisseria*, *Haemophilus*, and *Porphyromonas* and lower proportions of *Prevotella* and *Veillonella* compared with those in the Japanese population. Additionally, the geographical location had a remarkable effect on salivary microbiota, more than age, gender, or smoking, although the smoking status had a significant effect on the microbiome. In China, the salivary microbiome was dominated by 12 genera: *Streptococcus*, *Neisseria*, *Haemophilus*, *Prevotella*, *Porphyromonas*, *Veillonella*, *Gemella*, *Rothia*, *Granulicatella*, *Fusobacterium*, *Actinomyces*, and *Alloprevotella.* Among them, *Haemophilus* and *Prevotella* were the most abundant genera in healthy individuals (Wu et al., 2018).

In Yokohama in Japan, the FMD data showed that *Fusobacterium* was the main genus (**Table 1**). However, when the Japanese state or city was excluded, the main genus was *Rothia*. Mashima et al. (2019) analyzed the salivary microbiome in rural Thai children grouped into five clusters (east, west, south, north, and central) based on economic, food, and lifestyle factors and reported significant differences between *Veillonella* and *Prevotella* among the geographical regions (*p* < 0.05). In addition to their abundance, the presence of *V. parvula*, *R. aeria*, and *R. dentocariosa* indicated potential deterioration in oral hygiene, which also relates to dental caries history. Accordingly, individuals residing in Yokohama may exhibit comparatively better dental hygiene than the other populations. Also, *Prevotella* was the most common and abundant bacterium in East Asia (South Korea, Japan, China, and Thailand). Leake et al. (2016) reported the preponderance of eight major genera in the saliva samples of Swiss population: *Streptococcus*, *Neisseria*, *Prevotella*, *Haemophilus*, *Veillonella*, *Porphyromonas*, *Rothia*, and *Fusobacterium*. However, despite the geographic proximity between Germany and Switzerland, no common genera except *Fusobacterium* were found (Li et al., 2014). In contrast, Italians carried common genera (*Neisseria* and *Prevotella*).

Li et al. (2014) performed a comparative microbiome analysis of Alaskans, Germans, and Africans, including the Democratic Republic of Congo (*n* = 15), Sierra Leone (*n* = 13), and Uganda (*n* = 38) and revealed more similarities between Alaskans and Germans than Africans at the genus and OTU levels (Li et al., 2014). Both native Alaskans and Germans shared 13 common genera, while Alaskans and Africans shared only six genera (*Neisseria*, *Campylobacter*, *Granulicatella*, *Megasphaera*, *Selenomonas*, *Actinomyces*) and Germans and Africans carried three common genera (*Actinobacillus*, *Aggregatibacter*, and *Capnocytophaga*). Also, all of the foregoing populations shared only three genera (*Streptococcus*, *Fusobacterium*, and *Leptotrichia*) in common. Nasidze et al. (2009a) reported considerable differences in the diversity of the saliva microbiome between African populations, which were attributed to subsistence and dietary patterns. A study involving Sierra Leone and Congo, which are geographically distant but have similar dietary patterns, showed a higher degree of similarity with each other than with Batwa (Nasidze et al., 2009a).

Although no significant geographical signature of the salivary microbiome was detected in various populations, a frequency variation in the specific genera was found. For example, significant fluctuations in the frequency of *Enterobacter* were seen. *Enterobacter* constitutes approximately 28% of the sequences obtained from the Congo but not California, China, Germany, Poland, or Turkey. Furthermore, *Serratia* showed a relatively high frequency among Bolivians (Nasidze et al., 2009a). Murugesan et al. (2020) characterized the salivary microbiome of the Qatari population, which was associated with gender, aging, oral health, smoking status, and coffee or tea consumption. They found that Bacteroidetes, Firmicutes, Actinobacteria, and Proteobacteria are the common phyla, with Bacteroidetes being the predominant phylum, and at the genus level *Prevotella*, *Porphyromonas*, *Streptococcus*, and *Veillonella* (mean values of males and females, 54.3%, 8.1%, 6.6%, and 6.22%, respectively) were the most abundant in Qatari saliva samples. These results indicate that Qatar differed from countries where Firmicutes was the dominant phylum such as Bangladesh, UK, Japan, South Korea, and Brazil. Further sampling of various populations is required to demonstrate the unique geographic differences of each region.

## Vaginal Fluid

Identification of vaginal fluid in sexual assault cases is desirable for some forensic investigations. In particular, mixed samples containing vaginal fluid mixed with semen may suggest vaginal intercourse in sexual assault cases (Akutsu et al., 2012). Bacterial markers have been suggested to play a role in vaginal fluid identification based on the presence of *Lactobacillus* (87.5%), *Lachnospiraceae* (2.3%), *Prevotella* (1.1%), *Alcaligenaceae* (1.0%), *Erysipelatoclostridium* (0.9%), *Corynebacterium* (0.7%), *Peptoniphilus* (0.6%), *Bifidobacterium* (0.6%), *Anaerococcus* (0.5%), and *Staphylococcus* (0.5%) (Dobay et al., 2019). Also, *Subdoligranulum* (2.3%), *Blautia* (1.7%), *Escherichia-Shigella* (0.5%), *Anaerostipes* (0.4%), and *Stenotrophomonas* (0.3%) were found in the exposed vaginal fluid (Dobay et al., 2019). *Lactobacilli* play an important role in protecting the host from the urinary tract and genital infections and in maintaining the vaginal microbial balance; they occur predominantly in the vaginal microenvironment of healthy women (Boris et al., 1998; Mc and Rosenstein, 2000; Witkin et al., 2007). The abundance of *Lactobacillus* promotes acidic vaginal pH, which is the signature of *Lactobacillus* colonization, and is attributed primarily to the metabolism of glycogen to lactic acid (Mirmonsef et al., 2016; Das Purkayastha et al., 2019). However, vaginal pH and *Lactobacillus* diversity and dominance differ with individual lifestyles (Das Purkayastha et al., 2019). Recent studies and the human microbiome project reported nearly 60 vaginal microbiomes including four dominant species in the urinogenital tract: *L. crispatus*, *L. iners*, *L. gasseri*, and *L. jensenii* (Pavlova et al., 2002; Verhelst et al., 2004; Kroon et al., 2018).

**Figure 3:**
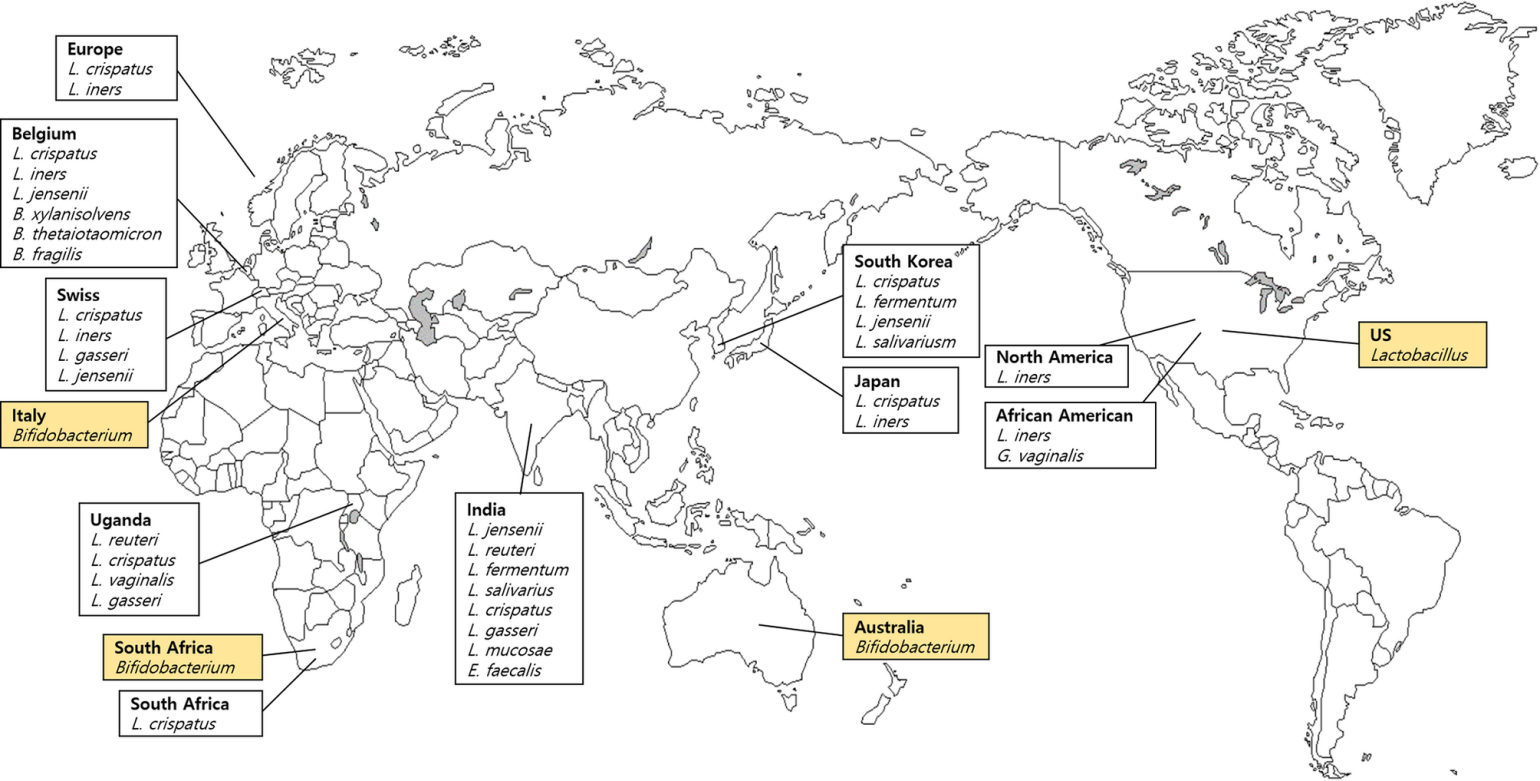
Dominant bacteria in vaginal fluid samples based on the Forensic Microbiome Database (www.fmd.jcvi.org) data and non-FMD studies. The yellow-colored box is from the FMD data, and only bacteria which account for over 10% are shown. Four countries from the FMD and 10 countries from previous studies were included, and the microbiome profiling was generated using 16S rRNA gene sequencing.

We analyzed seven vaginal fluid samples from four countries listed in FMD (**Table 1**) and 13 samples from 10 countries that are not included in the FMD (**Table 2**). Three body sites related to the vagina were listed in the FMD. Bacteria comprising less than 10% from vaginal introitus and mid-vagina from the USA were not listed. *Lactobacillus, Gardnerella* and *Shuttleworthia* were found in the vaginal fluid sample obtained from the United States. Surprisingly, *Lactobacilli* dominated the vaginal microenvironment and the *Bifidobacterium* was predominant in three countries (Australia, Italy, and South Africa), whereas *Holdemanella* constituted only a small proportion (**Figure 3**). Other species were not conspicuous in the UK. *Bifidobacterium* colonization occurred in the gastrointestinal tract, oral cavity, and vagina (Turroni et al., 2008; Arboleya et al., 2016; Gomez-Gallego et al., 2016).

Ravel et al. (2011) demonstrated the apparent differences in vaginal microbiome among the ethnic groups and reported that the vaginal microbiome of North American women (Caucasian, African American, Hispanic, and Asian) was dominated by *Lactobacillus* (59%–89%) (Ravel et al., 2011). Among four groups (Caucasian, African American, Hispanic, and Asian), the relative abundance of *Lactobacillus* among Hispanic and African American women was only 59.6% and 61.9%, respectively; however, it accounted for 80.2% of Asians and 89.7% among Caucasian women (Ravel et al., 2011). In addition, Hispanic and African American women carried an abundance of strictly anaerobic bacteria and *L. iners* was the predominant *Lactobacillus*. Another study of Caucasian and African American women from North America showed patterns similar to those reported by Ravel et al. (2011) (Zhou et al., 2010). In that study, vaginal communities of Caucasian and African American women were dominated by *Lactobacillus*, including *L. crispatus*, *L. iners*, *L. gasseri*, and *L. jensenii*. Furthermore, the prevalence of *Lactobacillus* was lower in African American women compared with Caucasian women (Zhou et al., 2010).

Verstraelen et al. (2016) analyzed 13 women from Belgium and showed similarities in microbiome composition involving *B. xylanisolvens*, *B. thetaiotaomicron*, and *B. fragilis* and an undetermined *Pelomonas* taxon constituted up to one-third of the endometrial microbiome. Also, six of 13 participants showed an abundance of *L. crispatus* or *L. iners*, characterized by the presence of the *Bacteroides* core (Verstraelen et al., 2016). Other studies of African Americans reported a number of *G. vaginalis* and bacterial vaginosis-associated bacterium-1 (BVAB1), and the diversity of the endometrial microbiome was significantly greater than in European women (Fettweis et al., 2014). The most prevalent species in African American women was *L. iners*, followed by *G. vaginalis*, BVAB1, and *L. crispatus*. In contrast, the most ubiquitous species in European females was *L. crispatus*, followed by *L. iners* and *G. vaginalis*. BVAB1, which was common in African American women, was only found in five of 416 European samples (Fettweis et al., 2014). *Lactobacillus* population in the healthy vaginas of Swedish women was studied by Vasquez *et al*. (Vasquez et al., 2002). The most predominant species were *L. crispatus*, *L. iners*, *L. gasseri*, and *L. jensenii*. Ugandans and South Africans showed similar trends in vaginal microbiome composition compared with Swedish (Jin et al., 2007; Pendharkar et al., 2013).

Studies investigating vaginal *Lactobacillus* composition were carried out among various Asian populations. In a comparative study of Korean and Ugandan women, five common genera were isolated: *Lactobacillus*, *Leuconostoc*, *Pediococcus*, *Streptococcus*, and *Weissella*. *L. fermentum* was detected only in Korean women, and *Pediococcus* was more common in Korean women (Jin et al., 2007). Furthermore, in Korean women, the most abundant species was *L. crispatus*, followed by *L. fermentum*, *L. jensenii*, *L. salivariusm*, *Pediococcus acidilactici*, and *Weissella kimchi*. The combination of *L. fermentum*, *P. acidilactici*, and *W. kimchi* was characteristic of the Korean population (Jin et al., 2007). In another study, *L. gasseri* was the predominant microbe among Chinese women, and with a higher prevalence in fertile women than in postmenopausal women (Zhang et al., 2012). Among the Japanese communities with *Lactobacillus* prevalence, *L. crispatus* was the predominant species, followed by *L. iners*, similar to Caucasian and African Americans in North America (Zhou et al., 2010). In India, several studies reported a geographic variation in vaginal microbiomes (Garg et al., 2009; Pramanick et al., 2017; Das Purkayastha et al., 2019): *L. jensenii* in the southwest region; *L. reuteri*, *L. fermentum*, and *L. salivarius* in the central region; and *L. crispatus*, *L. gasseri*, and *L. jensenii* in south India. Interestingly, the rare vaginal microbes *L. mucosae* and *Enterococcus faecalis* was found to be prevalent in Northeast India (Das Purkayastha et al., 2019). Das Purkayastha et al. (2019) reported a distinct and diverse vaginal microenvironment in various groups, ethnicities, and regions.

## Stool

The gut microbiota constitute the largest number of microbes compared with other body sites, and various microbiome studies have focused on the gut using the fecal samples (Lloyd-Price et al., 2016; Gupta et al., 2017). The composition and diversity of the gut microbiome are unique to individuals and are influenced by physical activity, diet, geographical environment, genetics, lifestyle, and ethnicity (Falony et al., 2016; Findley et al., 2016; Lynch and Pedersen, 2016; Zhernakova et al., 2016). Gut microbiota dysbiosis is attributed to metabolic and inflammatory diseases such as obesity, diabetes, and cancer (Adlerberth and Wold, 2009; Armougom et al., 2009). The most predominant phyla in the human gut are Bacteroides and/or Firmicutes, constituting more than 80% of the total microbiome (Lay et al., 2005). Dietary changes alter the composition of Bacteroides and Firmicutes (Clarke et al., 2012; Scott et al., 2013).

**Figure 4:**
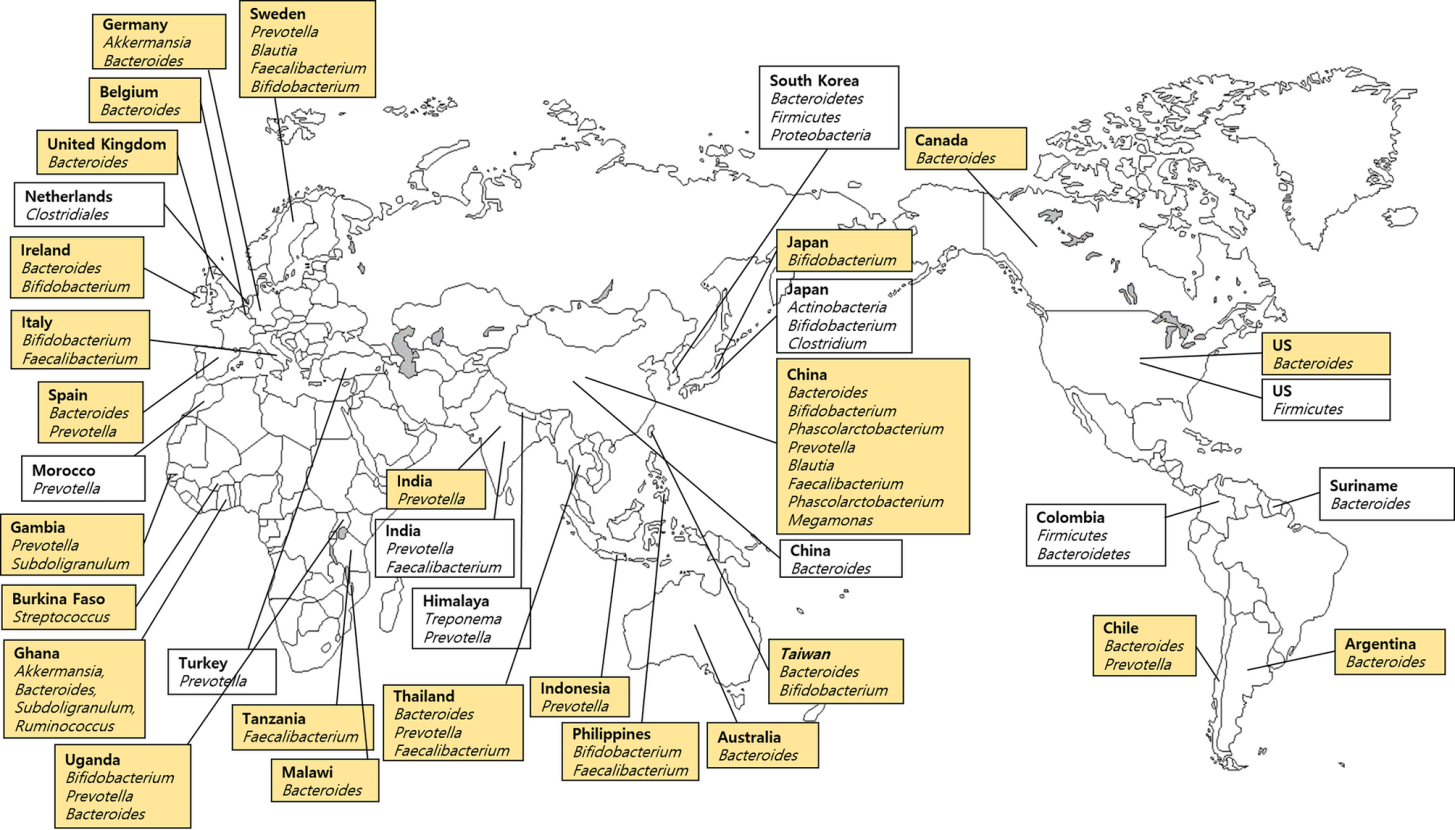
Dominant bacteria in stool samples based on the Forensic Microbiome Database (www.fmd.jcvi.org) data and non-FMD studies. The yellow-colored box is from the FMD data, and only bacteria which account for over 10% are shown. Twenty-five countries from the FMD and 11 countries from previous studies were included, and the microbiome profiling was generated using 16S rRNA gene sequencing.

Stool samples including 99 samples from 25 countries listed in the FMD (**Table 1**) and 10 samples from each of the 10 countries not included in the FMD were analyzed (**Table 2**). We divided the stool microbiome data reported in the FMD studies into five groups to facilitate analysis based on region and abundance (**Figure 4**). *Bifidobacterium* species were predominant in East Asia (Japan, China, Taiwan, and Philippines), *Prevotella* in South Asia (India, Thailand, and Indonesia), and *Bacteroides* was the most prevalent in West Europe (Germany, Belgium, UK, Ireland, and Spain), North and South America (USA, Canada, Chile, and Argentina) and Australia. The microbiome composition was inconsistent across African countries including Gambia, Ghana, and Tanzania.

In contrast, Koreans carried three phyla, which were predominantly found in stool microbiomes (Bacteroidetes, Firmicutes, and Proteobacteria) and accounted for a mean value of 99% of sequences (Shin et al., 2016). Also, Shin et al. (2016) reported that *Butyricimonas* of the phylum Bacteroidetes was predominant in Seoul (mainland), while *Catenibacterium* from the Firmicutes phylum was dominant in Jeju (an island). In addition, Japanese, Korean, and Chinese communities carried an abundance of Firmicutes, Actinobacteria, and Bacteroidetes, respectively (Nam et al., 2011). Japanese harbored predominantly *Bifidobacterium* and *Clostridium*, whereas Koreans carried *Prevotella* and *Faecalibacterium*, and Chinese had *Bacteroides* in their gastrointestinal microbiomes (Nam et al., 2011). Zhang et al. (2015) reported that *Phascolarctobacterium* of Firmicutes was the most abundant microbe in Chinese. In an Indian study, Tandon et al. (2018) analyzed urban cohorts derived from Western India and reported clear taxonomic differences in the microbiomes of American, Chinese, and Japanese populations. More than 80% of the sequences of gut microbiome derived from stool samples in the western Indian cohort belonged to five genera: *Prevotella*, *Faecalibacterium*, *Alloprevotella*, *Roseburia*, and *Bacteroides* (Tandon et al., 2018). These results were in line with FMD data which showed that *Prevotella* was the most predominant in the Indian urban population. A characteristic gut microbiome profile of the Himalayan population exhibited an increased abundance of *Treponema* and *Prevotella* and a decreased abundance of *Bacteroides* and *Bifidobacterium* (Jha et al., 2018). The difference across studies in the same country is attributed to sequencing methods, differences in sample preparation, bioinformatics, and cohorts from different ethnicities and provinces, especially in China.

The distribution of the microbial community in the stool samples is explained by the composition of the gastrointestinal microbiome. Compared with other samples, the stool samples tend to be affected by the habitat and dietary patterns. Gupta et al. (2017) reviewed the variation in the gastrointestinal microbiome based on the degree of urbanization. Models involved hunter-gatherers (Hadza of Tanzania, Pygmies of Central Africa, the Matses of Peru, and Amerindians of Venezuela), traditional farmers and fishermen (Bantus of Africa, the Tunapuco of the Andean highlands, or the rural Malawian communities), and urban industrialized populations (the USA and European) (Gupta et al., 2017). The findings suggest that the higher the transition from hunter-gatherers to urban population, the lower was the microbial diversity along with depletion of *Prevotella* and proliferation of *Bacteroides*. From a forensic perspective, access to the gastrointestinal microbiome may provide clues to the whereabouts of suspects based on a comparative analysis of the aforementioned studies involving the composition and diversity of stool samples and microbial clusters.

## Sequencing Platforms

It is important that the sample processing methods, data generation, and data analysis should be standardized for comparing microbiome data. Therefore, future investigations should reduce the differences in methodology and focus on standardizing analyses to ensure a better interpretation of the observed variation in these studies. Two sequencing approaches including 16S rRNA gene sequencing and whole genome shotgun sequencing have been used in the human-associated microbial analysis (Kuczynski et al., 2012). In this section, the strengths and limitations of 16S rRNA gene amplicons and shotgun metagenomics are reviewed.

Microbiome analysis based on 16S rRNA gene amplicons entails PCR amplification and sequencing of a variable region (or multiple regions). In contrast, shotgun metagenomics entails fragmentation and amplification of the microbiome genome, followed by sequencing (Ranjan et al., 2016). The primary microbial target is the 16S rRNA gene, which has been widely used. It is appropriate in the human/host DNA background (e.g., a skin swab) because the 16SrRNA primers only amplify the bacteria and archaea domains of life. The PCR step is appropriate for low biomass samples as it is inexpensive. However, single amplicon sequencing is limited by the differences in the relative abundances measured by bacterial community sequencing compared with the true relative abundances, and the primers used for amplification may misalign with the target region in some species. Human microbiome shotgun metagenomics can overcome these limitations, and theoretically, the entire genome of a microbe is amplified due to the additional targets for analysis. Furthermore, no amplification bias is detected since it bypasses primer-dependent PCR amplification. However, shotgun metagenomics entails higher sequencing costs and stochastic effects and generates a large number of uninformative reads that are not variable between taxa, which is a waste of sequencing efforts.

In contrast, recently, Schmedes et al. (2018) reported the use of hidSkinPlex, a panel of informative targets derived from skin microbiomes, which could be used with machine learning tools in human forensic applications. Clade-specific markers were investigated based on 286 bacterial (and phage) family-, genus-, species-, and subspecies-level markers, which were derived from publicly available datasets generated by human microbiome shotgun metagenomics (Oh et al., 2016). Also, the hidSkinPlex markers were used to classify skin microbiomes including three body sites (foot, hand, and manubrium). Based on supervised learning, all samples were correctly classified and the body site origin was estimated up to 86% accuracy. This preliminary approach yielded additional targets for analysis and interpretation after amplification. Furthermore, they provided insight into the utility of skin microbiome for human identification and may represent an appropriate balance between single-target and shotgun sequencing approaches.

Studies continue to examine and analyze samples more precisely. Recent studies have reported microbial community sequencing, including amplicon and shotgun metagenomic sequencing, for vaginal microbiome analysis (Berman et al., 2020). It is important to distinguish the different microbial species, since *Lactobacillus* and *Gardnerella* are the predominant genera in most of the vaginal microbiome in normal healthy women, especially when vaginal fluid is used as evidence (Ghemrawi et al., 2021). Likewise, microbial communities in each sample exhibit a characteristic distribution. Ethnic groups can be distinguished at the level of phylum or genus. Therefore, the accurate forensic application requires analysis at the taxonomic level based on the characteristic distribution of the microbial communities in each sample for optimal comparison. Whole microbial genomes obtained *via* shotgun metagenomic sequencing provide insight into functional genes and pathways of the microbiome, which cannot be obtained *via* 16S rRNA gene amplicon sequencing (Avershina et al., 2018; Berman et al., 2020). Furthermore, the poor resolution of 16S rRNA gene amplicon sequencing may not allow the identification of the location or ensure individualization of WGS (Gloor et al., 2017; Berman et al., 2020). In summary, although both methods have their pros and cons, shotgun metagenomics is more valuable in forensic applications, as differences between individuals might only be detected at the subspecies and strain level.

## Conclusion

This review provides insight into the differences in microbiome diversity and abundance among different samples of forensic interest (i.e., skin, saliva, vaginal fluid, and stool). The human microbiome, as a potential forensic biomarker(s), carries individual-specific information such as ethnicity or population affiliation, geographic location, lifestyle, as well as the microbiome origin in the tissue or body fluid, and human identification. Although the information provided by the FMD is relatively small, a comparative analysis with other studies yielded similar trends. In addition, previous studies revealed common bacterial species or phyla shared by microbiomes in nearby countries.

In the case of skin samples, *Staphylococcus* species were predominantly higher than *Corynebacterium* species in Asians compared with Americans. Based on both FMD and non-FMD studies, *Propionibacterium* was also found higher in North America. *Acinetobacter* and *Proteobacteria* were detected in Latin America. *Holdemanella* and *Fusobacterium* were characteristic of Korean and Japanese saliva samples, respectively. However, *Veillonella* and *Prevotella* were common bacteria shared by all Asians. *Veillonella* was unique and found only in Asians. However, the saliva microbiome of the Swiss population was more similar to the microbiome of Italians. According to Li et al. (2014), *Bacteroides* were not detected in 12 countries (Germany, Poland, Turkey, Georgia, China, Philippines, South Africa, Congo, Argentina, Bolivia, Louisiana, California) but was found only in native Alaskans (**Table 2** and **Figure 2**) (Nasidze et al., 2009b). *Serratia* and *Enterobacter* are vaginal fluid microbes endemic to Bolivia and Congo, respectively. *L. crispatus* and *L. iners* are predominant in all of the European countries in this study. *L. iners* and *L. crispatus* were dominant in America and Africa but not in Asia. Finally, in the case of the stool microbiome, which is the most abundant in the FMD, *Bacteroidetes* was detected in most countries. *Prevotella* was predominant in South Asian countries such as India, Himalaya, Turkey, Thailand, and Indonesia, whereas *Bacteroides* and *Bifidobacterium* were prevalent in East Asia including South Korea, Japan, and China. However, European, American, or African microbiomes showed no distinguishing features (**Table 2**; **Figure 4**).

Approximately equal numbers of bacterial and human cells are found in the human body, which provides novel and unique identifiable markers specific to the individual, even in identical twins (Voreades et al., 2014; Zhu et al., 2015). The variation in microbiome structure and composition may provide forensic evidence based on host lifestyle and pharmaceutical use (Gonzalez et al., 2016; Kuntz and Gilbert, 2017). A recent study highlighted the potential forensic applications based on geographic classification utilizing city-specific microbiomes (Danko et al., 2021). However, in reality, the available microbial databases are inadequate under various conditions (Hampton-Marcell et al., 2017). Also, several studies cannot provide a rationale supporting the use of microbial sequencing in forensic studies, probably because the microbiome changes over time in an individual (Oh et al., 2016). Therefore, while human microbial fingerprinting can never replace established traditional DNA profiling techniques, it is possible that in the future, it can reinforce the currently available tools used by forensic investigators. Further investigations are still required to demonstrate that microbial fingerprints can be used as effective evidence.

This review is the first study collating the available evidence and the observed variation in microbiomes from forensically relevant sources of human microbiome. As the FMD was also analyzed with 16S rRNA gene amplicon sequencing, we compared the signals under different geographies in different studies, which used the same technology/data type. However, if the 16S rRNA gene primer regions targeting each study differed, it is clearly a limitation in the comparison of microbiome studies. Also, these studies are likely to have different downstream bioinformatics pipelines with varying degrees of distribution at the genus level. Thus, comparative analyses are associated with limitations, which in turn have implications for comparisons involving body parts, body fluids, and geographic locations internationally.

Despite these limitations, we described the use of the FMD repository that contains human microbiome data from subjects across multiple countries. Additionally, we incorporated sequencing data from other independently published studies to understand the effects of body sites and geography on the human microbiome. Furthermore, the geographic location of microbial communities is critical to the development of innovative forensic techniques in similar comparative analyses using FMD and technologies based on 16S rRNA gene primers. Further studies are needed to determine the power and resolution of the microbial diversity and the abundance of robust forensic biomarkers using geographic location and lifestyle indicators. Therefore, it is anticipated that studies involving not only samples from skin, stool, vaginal fluid, and saliva but also urogenital, airway, ocular, and breast milk specimens will be analyzed to build a more representative cohort in Asians and Africans as well as Europeans and Americans.

## Author Contributions

H-WCho and Y-BEom participated in the design of the study, contributed to data reduction/analysis and interpretation of the results. H-WCho contributed to data analysis and interpretation of the results. All authors contributed to manuscript writing. All authors contributed to the article and approved the submitted version.

## Funding

This study was supported by the Soonchunhyang University Research Fund and a National Research Foundation of Korea (NRF) grant funded by the Korean government (MSIT) [NRF-2020R1F1A1071977].

## Conflict of Interest

The authors declare that the research was conducted in the absence of any commercial or financial relationships that could be construed as a potential conflict of interest.

## Publisher’s Note

All claims expressed in this article are solely those of the authors and do not necessarily represent those of their affiliated organizations, or those of the publisher, the editors and the reviewers. Any product that may be evaluated in this article, or claim that may be made by its manufacturer, is not guaranteed or endorsed by the publisher.
